# Cigarette Smoking and Adipose Tissue: The Emerging Role in Progression of Atherosclerosis

**DOI:** 10.1155/2017/3102737

**Published:** 2017-12-27

**Authors:** Zhiyan Wang, Di Wang, Yi Wang

**Affiliations:** Department of Cardiology, Shanghai General Hospital, School of Medicine, Shanghai Jiao Tong University, Shanghai 200080, China

## Abstract

Smoking is an established risk factor for atherosclerosis through several underlying pathways. Moreover, in the development of atherosclerotic plaque formation, obesity, defined as excess fat mass accumulation, also plays a vital role in dyslipidemia and insulin resistance. Substantial evidence shows that cigarette smoking induces multiple pathological effects in adipose tissue, such as differentiation of adipocytes, lipolysis, and secretion properties in adipose tissue. Therefore, there is an emerging speculation in which adipose tissue abnormality induced by smoking or nicotine is likely to accelerate the progression of atherosclerosis. Herein, this review aims to investigate the possible interplay between smoking and adipose tissue dysfunction in the development of atherosclerosis.

## 1. Introduction

Cardiovascular diseases (CVDs) are identified as the leading causes of death in many countries, in both developing world and industrialized regions [1], and these diseases include coronary artery diseases, ischemic stroke, and peripheral artery diseases. The basic pathology of the aforementioned diseases is the advancement of atherosclerosis (AS) leading to vascular stenosis and plaque rupture. Notably, it is estimated that approximately 11% of global cardiovascular deaths are attributed to smoking [1], indicating that smoking is one of the independent risk factors in AS. Recent papers have shown an increase in carotid artery intima-media thickness among currently smoking adolescents, which points to an early atherogenic remodeling of the vasculature in youth [2], further aggravating the global burden of disease. Therefore, a variety of studies have been dedicated to explore the underlying causes of smoking-induced atherogenesis.

Apart from cigarette smoking, obesity is another public health issue in that the worldwide prevalence of obesity has dramatically increased since 1980 [3]. Obesity, characterized as excessive adipose tissue, has a harmful effect on vascular function, and its associated comorbidities are prone to develop CVDs. Similar to smoking, obesity in childhood/adulthood and the long-term consequences of vascular injury can be clinically relevant [4]. The underlying mechanisms of adipose tissue in AS have been studied in recent years, while the accurate pathways remain to be elucidated.

In particular, in 2002, it has estimated that approximately 20% of US smokers, about 9 million people, were obese [5]. Several studies have reported that people with coexistence of obesity and current smoking habits show especially large risks for mortality related to CVDs and other diseases [6, 7]. Furthermore, a burgeoning body of studies has reported that cigarette smoking has a complicated effect on body weight and the function of adipose tissue [8–10]. Therefore, this specific interaction between cigarette smoking and adipose tissue in atherogenesis may represent a crucial target for future therapy. The objective of the present paper is to delineate the mechanisms through which exposure to chemicals in cigarette smoking affects the differentiated status and functions of adipocytes, which may contribute to AS.

## 2. Pathogenesis of Atherosclerosis: a Brief Overview

Accrued data have defined atherosclerosis as a chronic low-grade inflammation of the vasculature system characterized by atherosclerotic plaque formation and rupture. Abnormal accumulation and retention of low-density lipoprotein (LDL) and lipoprotein remnants have been implicated as initial triggers [11]. Associated enzymes in the vessel wall have the ability to modify this LDL to oxidized LDL, which serve as inflammatory signals [12]. Inflammatory cells are subsequently recruited to the arterial wall, such as monocytes, which differentiate into macrophages and are, as a result, activated to engulf oxidized LDL via scavenger receptors, creating foam cells, which secrete chemokines and other kinds of cytokines that further create a vicious cycle, more immune cell infiltration, and activation [13]. Additionally, there is another possible pathway in which many of these lipid-rich macrophages undergo apoptosis and necrosis, releasing their contents into the extracellular space and then formatting a necrotic core. Proliferation and migration of vascular smooth muscle cells (VSMCs) also participate in the pathological process of plaques, resultantly stimulating the release of cytokines, such as interleukin-1, 8 (IL-1, 8) and interferon-*γ* (IFN-*γ*). Collectively, these various cascade reactions lead to fatty streak formation and followed advancement of plaques. Aside from immune cell entry into the plaque through the intima, immune cells are also observed in the outer part of the vessel wall, the adventitia, and perivascular adipose tissue (PVAT). Of note, collagenous conduits and vasa vasorum may bridge the communication between the intima and adventitia, which highlights the role of the adventitia in coordinating the immune response in AS [14]. Furthermore, due to absence of the fascia barrier between PVAT and adventitia, it seems possible that PVAT secretes various kinds of local adipokines and cytokines to interact with the adventitia [15]. As a result, PVAT and the adventitia have provided emerging insight on atherogenesis.

## 3. Smoking and Atherosclerosis: a Well-Known Mechanism

**Figure 1:**
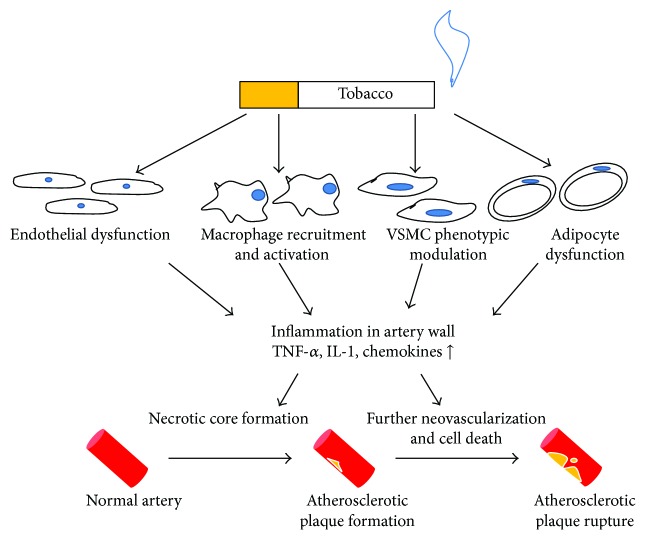
Smoking-associated inflammatory response in artery walls. Smoking causes endothelial dysfunction with VSMCs phenotypic modulation. In addition, more macrophages are recruited and activated to secrete cytokines and chemokines. The resultant inflammatory response leads to atherosclerotic plaque formation and subsequent plaque rupture. TNF-*α*: tumor necrosis factor-*α*; IL-1, interleukin-1.

Cigarette smoke contains more than 4000 different components, which complicates the understanding of the potential mechanisms of tobacco-related diseases [16]. Among these constituents, nicotine has been identified as one of the most important ingredients that participate in vascular inflammation. Nicotine has been shown to increase physiological parameters, such as blood pressure and heart rate [17]. In addition, while binding with high-affinity nicotinic acetylcholine receptors (nAChRs), nicotine exerts several bioactive actions on different cellular effectors involved in plaque formation and progression [18]. According to a number of in vitro and clinical studies, there is strong evidence that exposure to cigarette smoking impairs the normal prosperity of endothelial cells, especially in the youth group [19]. Nicotine and the resulting increased oxidative stress induce vascular endothelial dysfunction via inhibition of the activation of endothelial nitric oxide synthase (eNOS) and decreasing the generation and bioavailability of nitric oxide (NO) [20]. Moreover, nicotine increases the expression of adhesion molecules on endothelial cells, namely, intracellular adhesion molecular-1 and E-selectin, as a result of enhanced attachment and transmigration of monocytes to the vessel wall [21]. Considering the types of inflammatory cells, a recent study has shown that nicotine upregulates CD36 expression in monocytes/macrophages via activation of nAChRs, facilitating to engulf the lipid particles by macrophages [22]. Macrophages stimulated by the treatment of nicotine secrete elevated inflammatory cytokines, namely, tumor necrosis factor-*α* (TNF-*α*), IL-1*β*, and chemokines, creating the proinflammatory microenvironment in the subendothelium [23]. Furthermore, it is shown that VSMCs undergo the contractile-to-synthetic transition, characterized by enhanced growth and migration of VSMCs, which contributes to foam cell formation [24]. Apart from the above alternations of cells, other substances and structures can be induced upon exposure of nicotine. For instance, under the treatment of nicotine, vasa vasorum has been shown to expand to intima plaque and the neovasculature is discovered in the plaque, one of the markers of the instable plaques [18]. Finally, exposure to smoking results in platelet activation, stimulation of a coagulation cascade, and impairment of anticoagulative fibrinolysis, which, in turn, promotes pathological thrombus formation [25]. Overall, cigarette smoking or nicotine has multiple actions on the advancement of AS (see Figure 1).

Polycyclic aromatic hydrocarbons (PAHs), another class of compounds in cigarette smoke, also induce atherogenesis [26]. PAHs binding with aryl hydrocarbon receptor can downregulate the cholesterol efflux [26]. Other cigarette-derived substances also participate in various pathways to promote AS [27].

## 4. Adipose Tissue and Atherosclerosis

Obesity is characterized by excessive or abnormal accumulation of adipose tissue. The traditional roles of adipose tissue are to store free fatty acids after eating and release them in fasting state, which is sensitive to the regulation of insulin. Notably, it is now widely recognized that adipose tissue is not only a storage depot but also an active source of bioactive factors, such as adipokines, which influence lipid levels, inflammation, oxidative stress, insulin resistance, and AS [28]. There are various kinds of adipokines participating in the advancement of atheromas, the bioactive actions of which are shown in Table 1.

Accumulating evidence indicates that obesity leads to adipose tissue dysfunction, including adipocyte hypertrophy, enhanced inflammation, and impaired vascular structure and function [29]. In light of the wide distribution of adipose tissue in human bodies, adipose tissue has the ability to exert systemic effects on the cardiovascular risk factors and associated CVDs. Blood lipid abnormalities caused by excess adipose tissue are classical features of metabolic syndrome, such as the presence of small dense LDL particles, which in part induce the proinflammatory place within the vascular wall [30]. More importantly, adipose tissue dysfunction leads to an imbalance in the production of adipokines. When obesity is present, the main characteristics of adipokine concentration are identified as elevated levels of leptin, resistin, and TNF-*α* [31–33], in conjunction with a decline in production of adiponectin and omentin [34, 35]. According to Table 1, the imbalance of adipokines relevant to obesity would, upon interaction with multiple vascular cells, deteriorate the formation and advancement of plaques. Additionally, adipose tissue inflammation appears to be of importance in AS. For example, patients with coronary artery diseases produce higher levels of proinflammatory cytokines (such as TNF-*α*, IL-6, and visfatin) in epicardial adipose tissue [36]. Additionally, obese adipose tissue contains more M1 type macrophages, mast cells, and neutrophils, through in situ proliferation and migration, thus augmenting proinflammatory responses [37]. Adipocytes seem to associate with certain immune cells through cytokine secretion and antigen presentation [37], which may synergistically accelerate the chronic inflammation in obese subjects.

Aside from systematic effects, adipose tissue may have crucial local actions in AS due to unique types of adipose tissue, namely, PVAT. Both visceral adipose tissue and PVAT are mainly made up of white adipose tissue, which is more relevant to metabolic syndrome. Functionally, it is widely accepted that PVAT has a mechanical role as a connective tissue to protect the vessels against adjacent tissue [38]. In addition, PVAT has been reported to produce a wide range of adipokines, similar to visceral adipose tissue to interact with correspondent intima and meditate the vascular inflammation. Of note, compared to adipocyte in other adipose tissues, PVAT adipocytes release more angiogenic factors including thrombospondin-1, CC-chemokine ligand 2 (CCL2), and hepatocyte growth factor to mediate vascular remodeling [39]. Finally, the vasa vasorum serves as a conducting tube that delivers blood components, local adipokines, and inflammatory cells from PVAT, which highlights the pathological characters of PVAT in the atherosclerotic process.

## 5. Link between Cigarette Smoking and Adipose Tissue in Atherogenesis

### 5.1. Nicotinic Receptors in Adipose Tissue

nAChRs are members of a family of ligand-gated, pentameric ion channels that are tightly arranged around a central pore [18]. nAChRs are divided into muscular (*α*1, *β*1, *γ*/*ε*1, and *δ*1) and neuronal AChRs (*α*2–*α*9 and *β*2–*β*4) [40]. Traditionally, the major biological force of these receptors is to mediate the effects of the endogenous neurotransmitter, acetylcholine, at neuromuscular junctions. In addition, nonneuronal cells may also express functional nAChRs. Apart from the above cells, several lines of evidence show that exogenous nicotine and other components of cigarette smoking have the ability to bind to high-affinity nAChRs on multiple cell types in the cardiovascular system, specifically endothelial cells and VSCMs, which thereby exert direct actions and other cellular effectors that participate in the atherosclerotic plaque formation and growth [18, 27]. Furthermore, the study by Liu et al. detected nAChR expression in adipocytes by a reverse transcriptase-polymerase chain reaction. Under the further analysis of subunits for nAChRs, they found *α*1-7, 9, 10, and *β*1-4 mRNAs expressed in adipocytes. Cancello et al. evaluated *α*7-nAChR expression levels in whole subcutaneous adipose tissue obtained from morbidly obese subjects and from normal weight healthy individuals, further indicating that this receptor modulates inflammatory gene expression in human adipocytes [41]. The existence of *α*7-nAChRs in adipose tissue may establish a potential connection between nicotine and adipose tissue in chronic low-grade inflammation, thereby affecting the course of AS. Accordingly, several basic studies are employed to clear out the accurate signal pathway underlying the bind with nicotine and nAChRs in adipocytes. For instance, Wu et al. suggested that nicotine has the capacity to react with white adipose tissue through *α*7-nAChRs and adenosine 5′-monophosphate-activated protein kinase (AMPK) [8]. It follows that *α*7-nAChRs play a fundamental role in the nicotine-induced abnormality within adipose tissue.

Apart from nAChRs, there is another receptor located on the surface of adipocytes, local *β*-adrenergic receptor, to stimulate lipolysis in adipose tissue by systemic infusion of nicotine [42]. Several chemicals mediate the nicotine effects on *β*-adrenergic receptor, such as the catecholamines epinephrine and norepinephrine and other metabolites. The nicotine-evoked catecholamine release in the brain tissue, such as the striatum and hypothalamus [43], is possibly mediated by *β*2 and *β*4 nAChRs [44]. The nicotine metabolite nitrosamine 4-(methylnitrosamino)-1-(3-pyridyl)-1-butanone, for example, can directly bind with *β*-adrenergic receptor to induce lung cancer, pancreatitis, and endothelial cell injury [45–47]. In both brown and white adipocytes, Cao et al. have found that there is the *β*-adrenergic/cAMP/PKA signaling pathway, participating in brown fat thermogenesis [48]. Furthermore, data from Fasshauer et al. shows that in 3T3-L1 adipocytes, *β*-adrenergic stimulation exerts certain effects on the physical function of adipocytes [49]. Thus, the above interactions may bridge the connection between nicotine or nicotine metabolites and adipose tissue. However, the downstream effects of this signaling pathway remain elusive, lacking the direct evidence needed to determine the accurate mechanisms.

### 5.2. Alterations in Adipocyte Differentiation and Functions of Adipose Tissue

#### 5.2.1. Smoking and Adipogenesis

**Figure 2:**
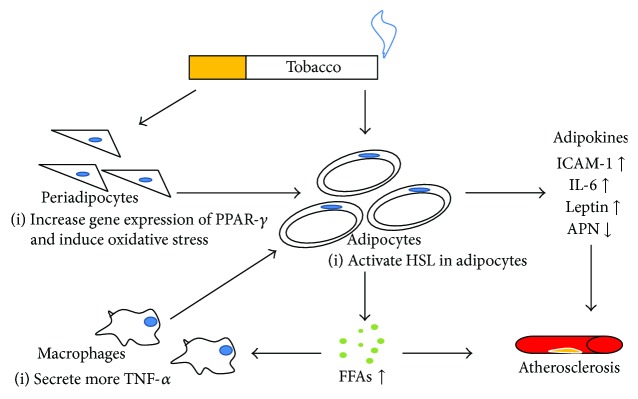
The direct effects of smoking on adipocytes. Smoking has direct actions on the differentiation of adipocytes. On the other hand, smoking can promote the release of FFAs through HSL. In turn, increased FFAs can stimulate macrophages to produce more TNF-*α*, which further induces adipocytes to secrete various kinds of adipokines, such as ICAM-1, IL-6, and leptin. In the above process, several products (FFAs and adipokines) can influence the artery walls. PPAR-*γ*, peroxisome proliferator activated receptor-*γ*; HSL, hormone-sensitive lipase; FFAs, free fatty acid; ICAM-1, intracellular adhesion molecule-1; IL-6, interleukin-6; APN, adiponectin.

In many populations, cross-sectional studies show that mean body mass (BMI) tends to be lower among smokers than nonsmokers. The underlying reason is the increase in metabolic rate induced by cigarette smoking. However, when using the waist circumference or waist-to-hip ratio instead of BMI, there is evidence suggesting that cigarette smoking is in favor of greater accumulation of visceral fat [50]. A rat experiment showed that maternal exposure to nicotine during lactation may promote obesity in adulthood, accompanied with higher central adiposity and hyperleptinemia [51]. An in vivo study using Sprague-Dawley rats shows that prenatal nicotine exposure led to an increase in epididymal white adipose tissue weight at weaning, and marked hypertrophy of adipocytes, with increased gene expression of proadipogenic transcription factors such as peroxisome proliferator-activated receptor-*γ* (PPAR-*γ*), resulting in increased body weight and fat deposition [52]. PPAR-*γ* is widely considered to be essential in inducing differentiation from preadipocytes to mature adipocytes. In one animal study, the supraphysiological activation of PPAR-*γ* by troglitazone, a kind of PPAR-*γ* agonist, increases the number of small adipocytes, which in turn promotes a flux of free fatty acids (FFAs) from the liver and muscle into WAT, leading to the upregulation of insulin sensitivity at the expense of increased WAT mass [53]. This is consistent with other studies that have reported that PPAR-*γ*-deficient mice are protected against adipocyte hypertrophy and obesity induced by high-fat diet and aging [54]. Of particular interest, cells with a reduction in PPAR-*γ*2 expression caused by artificial zinc finger repressor proteins are unable to undergo adipogenic differentiation, thereby suggesting that PPAR-*γ*2 plays a central role in orchestrating the adipogenesis process [55]. Of note, PPAR-*γ* has been reported to have anti-inflammatory activity, but the specific role in adipocyte remains unclear. Given that the possible elevated expression of PPAR-*γ* is induced by nicotine, more research is needed to elucidate the “nicotine-PPAR-*γ*” axis in the development of adipogenesis (Figure 2).

In light of the underlying pathways to influence the activity of PPAR-*γ*, it is plausible that oxidative stress may have a certain role in mature adipocyte formation. Exposure of 3T3-L1 adipocytes to concentrations of nicotine ranging from 60 nM to 6 *μ*M significantly increased the generation of reactive oxygen species (ROS) [56]. Additionally, female C57BL/6 mice, upon exposure to acute smoking showed increased activity of glutathione peroxidase in the inguinal adipose tissue, potentially proving that oxidative stress was increased in adipose tissue [57]. Another model using *α*7-nAChR-specific lentivirus shRNA showed similar results [8], which emphasized the involvement of nAChRs on ROS production. Additionally, Lee et al. reported that ROS facilitate the cellular differentiation from 3T3-L1 preadipocytes to adipocytes by accelerating the mitotic clonal expansion, the second course of adipocyte differentiation, whereas an antioxidant treatment causes S-phase arrest [58]. Thus, nicotine may, in part, induce oxidative stress in adipose tissue contributing to the mature adipocyte.

Other substance from cigarette has also been investigated in several studies. In epididymal fat, chronic carbon monoxide also led to a significant decrease in adipocyte size and an increase in adipocyte number [59]. Additionally, through culturing the isolated adipocytes from mice adipose tissue, it was found that PAHs had the ability to increase the adipose tissue mass [60]. This may attribute to the PPAR activation of PAHs and their metabolites [61]. These adipogenesis disturbances may cause the abnormality of adipose tissue, such as enhanced lipolysis and dysfunction of adipokines secretion, which directly promotes cardiovascular diseases and worsen metabolic diseases and related risk factors, as Bays reviewed in detail [62].

#### 5.2.2. Smoking and Lipid Metabolism

Exposure to cigarette smoke may break the balance of lipid levels through affecting the function of adipose tissue. This impaired state is commonly referred to as “dyslipidemia” and is considered to be the putative link between smoking and AS.

Considering smoking-induced dyslipidemia, the regulation of lipolysis in adipose tissue is the key event. Both clinical and animal studies found that nicotine can block phosphodiesterase activation to promote lipolysis and as a result increase the levels of circulating FFAs [8], which is consistent with the results from An et al. in 2007 [56]. Of great significance, intracellular enzyme hormone-sensitive lipase (HSL), which participates in the hydrolysis of triglyceride in adipocytes, is probably regulated by treatment of nicotine. *β*-Adrenergic receptor stimulation by catecholamines increases the level of cAMP and, ultimately, activates HSL [63]. Based on this, it is conceivable that via *β*-adrenergic receptors, smoking has an effect on the activation of HSL, producing elevated FFAs, which are synthesized into triglycerides through transportation into the liver and then secreted back into circulation as triglyceride-rich lipoprotein, forming the profile of dyslipidemia (Figure 2). In contrast, one kind of PAHs, benzo[a]pyrene, has a significant inhibitory effect on epinephrine-induced FFAs release [60]. It meant the complexity of the compounds of cigarette smoking, which need further studies to explore out.

#### 5.2.3. Smoking and Endocrine Function of Adipose Tissue

As previously discussed, adipokines and cytokines secreted by adipose tissue participated in the inflammation within the artery wall. Recently, the aim of many studies is to investigate how cigarette smoking affects the secretion of adipokines, thus resulting in a deeper understanding of the relationship between smoking and adipose tissue mediated by adipokines in the advancement of atherosclerotic plaques. Next, we will explore the alterations of many crucial adipokines under exposure to cigarette smoke, such as adiponectin, TNF-*α*, and leptin, as well as adipose tissue inflammation.

*(1) Adiponectin and TNF-α*. Hypoadiponectinemia was often detected in smokers without sexual difference, which is an independent risk factor for diabetes and AS [64, 65]. In accordance with this, adiponectin levels in healthy Greek smokers were elevated 9 weeks after smoking cessation [66]. In 3T3-L1 adipocytes, Iwashima et al. found that incubation with nicotine significantly reduced adiponectin mRNA expression and adiponectin secretion with dose dependence [64]. Moreover, cultured adipocytes and the adipose tissue of wild-type mice exposed to cigarette smoke extract high-molecular-weight adiponectin and subsequently block its release [67], suggesting an inverse association between smoking and the level of adiponectin.

An increasing body of studies further investigates the potential mechanisms underlying smoking-induced low adiponectin concentration. Of great interest, data from *β*2^−/−^ mice models have shown that the *β*2 nAChR subunit may reduce the expression levels of AdipoQ genes under chronic nicotine administration [68], thus decreasing the generation of adiponectin. The exact signaling pathway involved in this process, however, has yet to be fully elucidated. Moreover, the activation of *β*-adrenergic receptors might have similar effects on the production of adiponectin, via a marked depletion of tissue adiponectin mRNA and elevated secretion of immature 30 kDa form [69]. Research has shown that cAMP, the second messenger of *β*-adrenergic receptors, may act indirectly through enhanced synthesis of inhibitory protein to destabilize adiponectin mRNA [69].

Of note, it is widely recognized that there is a negative interaction between adiponectin and TNF-*α*, investigated by in vivo study from Maeda et al. [70]. In human epicardial adipose tissue, tobacco smoking induces elevated levels of TNF-*α* and IL-6, creating the proinflammatory profile [71]. In vitro experiments have shown that TNF-*α* stimulates nuclear factor-kappa B (NF-*κ*B), PI-3 kinase, and jun-N-terminal kinase cascades, subsequently enhancing lipolysis in periadipocytes [72]. In contrast, nicotine was detected to reduce TNF-*α* production from rat adipocytes in a dose-dependent manner via nAChRs, while the underlying mechanism remains to be clarified [68, 73]. Consequently, further research should pay more attention to the nAChRs in adipocytes and adipose tissue macrophages, both of which are the main source of TNF-*α* and adiponectin.

*(2) Leptin*. Results from several studies show that nicotine has paradoxical effects on the secretion of leptin among smokers. As a result of maternal exposure to nicotine during lactation, offspring rats displayed the hyperleptinemia phenotype and higher visceral and total body fat mass [51, 74]. In contrast, previous studies reported a negative trend between cigarette smoking and plasma leptin levels in both diabetic subjects and healthy subjects [75]. After BMI adjustment, smokers were found to have lower leptin concentrations, which is probably attributed to the indirect effects of elevated catecholamine levels, rather than nicotine [76]. Of particular significance, Perkins et al. found no difference in leptin concentration on smoking status after controlling for BMI and age, while leptin levels only in women increased after smoking cessation [77]. One putative cause for these different conclusions may be the lack of attention to ethnic differences and the varying quantity of cigarettes. More research is needed to elaborate on the association between leptin and smoking/nicotine.

*(3) Adipose Tissue Inflammation*. As previously discussed, adipose tissue inflammation plays a fundamental role in the progression of atherosclerotic plaques. Both adipose tissue macrophages and T lymphocytes also contribute to formation of the inflammatory microenvironment in adipose tissue.

Interestingly, a large number of studies focus on the interplays between smoking and lymphocytes, so there is an emerging notion that smoking may mediate the numbers and functions of lymphocytes and the inflammation status within adipose tissue. According to existing research, it primarily utilizes the following pathways in the aforementioned pathological process.

First, certain cytokines and adipokines induced by smoking might influence the number and function of resident lymphocytes in adipose tissue, as well as local inflammation. For example, after activation of the NLRP3 inflammasome, enhanced CCL2 expression in perivascular adipocytes of smokers is previously discussed [78]. Notably, the stimulation by smoking or nicotine on macrophages was shown to further trigger adipose tissue inflammation. Through the Toll-like receptor 4 (TLR4), cigarette smoke induces phosphorylation of NF-*κ*B in cultured macrophages to activate inflammatory signaling, resulting in more cytokines secretion [79]. Aside from these direct effects, increased FFAs on exposure to smoking can be recognized by TLR-4 to exert indirect actions. As shown in Table 1, adiponectin and leptin also participate in the phenotype transition, such as macrophage polarization and T lymphocytes [80, 81]. Hence, adipose tissue inflammation will in turn exacerbate the adventitia response and eventually deteriorate intima injury. Given these smoking-induced changes in adipose tissue, it is important to identify whether smoking is directly involved in the recruitment and activation of lymphocytes or not and what the potential mechanisms of involvement are.

## 6. Conclusion

There is a complex yet significant interaction between cigarette smoking exposure, adipose tissue, and atherosclerotic plaque formation and rupture. Smoking or nicotine appears to affect the differentiation and functions of adipocytes, as well as the inflammatory status in adipose tissue. The current literature has always shed light upon the multiple molecular mechanisms by which components of tobacco smoke can initiate endothelial injury. Of great interest is the complicated interaction between smoking and adipose tissue and how this could establish a better understanding of the course of smoking-related AS. However, there are few studies that provide direct evidences responsible for the effects on adipose tissue by nicotine. Therefore, as the knowledge of nicotine-induced dysfunction in adipose tissue has advanced, it has become clear that there exists a “nicotine-adipose tissue-AS” axis, which paves the way for the development of further targeted therapy.

## Figures and Tables

**Table 1:** Adipokines and their bioactive functions associated with cardiovascular diseases.

Adipokine	Bioactive functions associated with cardiovascular diseases	Reference
Leptin	Increase heart rate and elevate blood pressure level	[82]
	Increase lipolysis in skeletal muscle and adipocytes	[83]
	Increase reactive oxygen species secretion in endothelial cells, such as H_2_O_2_ and HO generation	[84]
	Facilitate cholesterol accumulation in macrophages	[85, 86]
	Promote the expression of matrix metallopeptidase-2 in VSMCs	[87]
	Act on various types of immune cells to promote the release of proinflammatory cytokines	[81, 88]

Adiponectin	Reduce tissue triglyceride content and upregulate insulin sensitivity	[89]
	Suppress endothelial cell apoptosis	[90]
	Suppress TNF-*α*-induced NF-*κ*B activation to decrease the recruitment of monocytes	[91]
	Inhibit the expression of scavenger receptors-A1 of macrophages and mediate polarization toward anti-inflammatory M2 phenotype	[80, 92]
	Attenuate proliferation and migration of VSMCs	[93]

TNF-*α*	Downregulate insulin resistance	[94]
	Increase expression of adhesion molecules	[95]
	Induce the migration and proliferation of VSMCs	[96]

Omentin	Proangiogenic property and inhibition of vascular inflammation	[97, 98]
	Promote NO production and its vasodilating effect of vascular	[99, 100]

Resistin	Subsclinical marker of atherosclerosis	[101]
	Increase the levels of endothelin-1, VCAM-1, and CCL2	[102]
	Promote foam cell formation by the dysregulation of scavenger receptors in macrophages	[103]

A-FEBP	The major mediator of vulnerable plaque formation	[104]
	Secrete more proinflammatory cytokines, such as TNF-*α* and CCL2	[105]

Chemrin	Magnify the functions of adhesion molecules	[106]

VSMCs, vascular smooth muscle cells; TNF-*α*, tumor necrosis factor-alpha; NF-*κ*B, nuclear factor-kappa B; NO, nitro oxide; VCAM-1, vascular cell adhesion molecule-1; CCL2, CC-chemokine ligand 2; A-FABP, adipocyte fatty acid binding protein.

## Abbreviations

AS: Atherosclerosis
AMPK: 5′-Monophosphate-activated protein kinase
BMI: Body mean index
CVDs: Cardiovascular diseases
cAMP: Cyclic adenosine monophosphate
FFAs: Free fatty acids
HSL: Hormone-sensitive lipase
IL: Interleukin
IFN: Interferon
LDL: Low-dense lipoprotein
nAChRs: Nicotinic acetylcholine receptors
NF-*κ*B: Nuclear factor-kappa B
PPAR-*γ*: Peroxisome proliferator activated receptor-*γ*
PVAT: Perivascular adipose tissue
ROS: Reactive oxygen species
TLR4: Toll-like receptor 4
TNF: Tumor necrosis factor
VSMCs: Vascular smooth muscle cells.
